# Automatic Lugano staging for risk stratification in lymphoma: a multicenter PET radiomics and machine learning study with survival analysis

**DOI:** 10.1097/MNM.0000000000002046

**Published:** 2025-09-03

**Authors:** Setareh Hasanabadi, Seyed Mahmud Reza Aghamiri, Ahmad Ali Abin, Maryam Cheraghi, Mehrdad Bakhshayesh Karam, Habibeh Vosoughi, Farshad Emami, Hossein Arabi

**Affiliations:** a Department of Medical Radiation Engineering; b Faculty of Computer Science and Engineering, Shahid Beheshti University; c Shahid Beheshti University of Medical Sciences, National Research Institute of Tuberculosis and Lung Diseases; d Nuclear Medicine Department, Masih Daneshvari Hospital; e Research Center for Nuclear Medicine, Tehran University of Medical Sciences, Tehran; f Razavi Cancer Research Center, Razavi Hospital, Imam Reza International University, Mashhad, Iran; g Division of Nuclear Medicine and Molecular Imaging, Geneva University Hospital, Geneva, Switzerland

**Keywords:** automatic Lugano staging, lymphoma, machine learning, PET radiomics, risk stratification, survival analysis

## Abstract

**Background:**

Lymphoma staging plays a pivotal role in treatment planning and prognosis. Yet, it still relies on manual interpretation of PET/computed tomography (CT) images, which is time-consuming, subjective, and prone to variability. This study introduces a novel radiomics-based machine learning model for automated lymphoma staging to improve diagnostic accuracy and streamline clinical workflow.

**Methods:**

Imaging data from 241 patients with histologically confirmed lymphoma were retrospectively analyzed. Radiomics features were extracted from segmented lymph nodes and extranodal lesions using PET/CT. Three machine learning classifiers (Logistic Regression, Random Forest, and XGBoost) were trained to distinguish between early-stage (I–II) and advanced-stage (III–IV) lymphoma. Model performance was evaluated using area under the curve (AUC), sensitivity, specificity, and accuracy together with survival analysis.

**Results:**

Among the three models evaluated, the logistic regression model incorporating both nodal and extranodal radiomic features performed the best, achieving an AUC of 0.87 and a sensitivity of 0.88 in the external validation cohort. Including extranodal features significantly improved classification accuracy compared to nodal-only models (AUC: 0.87 vs. 0.75). Survival analysis revealed advanced-stage patients had a fourfold higher mortality risk (hazard ratio: 0.22–0.26, *P* = 0.0036) and a median survival of 84 months. Key radiomic features, such as tumor shape irregularity and heterogeneity, were strongly associated with staging, aligning with Lugano criteria for extranodal spread.

**Conclusion:**

This study demonstrated the potential of PET radiomics features for automated Lugano staging. Adding extranodal features significantly improved staging accuracy and informed treatment.

## Introduction

Lymphoma, which accounts for about 5% of all cancers, is the most common type of blood cancer [1]. Accurate staging is essential to guide treatment decisions and predict patient outcomes. Staging not only defines the anatomical extent and location of disease, but also provides prognostic information, enables comparisons across clinical studies. Currently, PET/computed tomography (CT) imaging is the gold standard for staging patients with lymphoma [2,3], but visual interpretation of the images still relies on individual judgment.

Although PET/CT is the gold standard in hospitals for lymphoma staging, it requires visual interpretation by a physician, which results in variation between physicians and centers, making consistent and reliable staging difficult [2].

Recent advancements in radiomic techniques, with or without the integration of machine learning, utilize extensive quantitative data from PET/CT imaging. These techniques have shown promise in lymphoma management, with most studies focusing on predicting treatment response and patient outcomes [4–29]; however, these efforts have shown promise in supporting personalized medicine, relatively little attention has been given to radiomics-based approaches for initial staging in lymphoma.

Leveraging radiomics to standardize and potentially improve this process represents an important but underexplored opportunity. As an alternative to visual interpretation for staging, radiomics combined with machine learning enables a quantitative and reproducible analysis of PET images [30–34].

In this study, we aimed to develop a harmonized approach for distinguishing early-stage from advanced-stage lymphoma using multicenter PET radiomics and machine learning. We also evaluated whether radiomics features extracted only from nodal lesions are sufficient for staging, or if adding extranodal features improves classification performance. To assess clinical relevance, survival analysis was performed across the stages.

## Methods

### Patient demographics and study design

The retrospective cohort study was carried out at two centers: the main center (Masih Daneshvari Hospital, Tehran, Iran) from 2014 to 2024 and a secondary center (Razavi Hospital, Mashhad, Iran). The study was approved by the Medical Ethical Review Committee of Shahid Beheshti University of Medical Sciences under ethical code IR.SBMU.NRITLD.REC.1402.060, and as it was noninterventional, the committee waived informed consent for patients with an initial diagnosis of lymphoma who had a baseline fluorine-18 fluorodeoxyglucose (^18^F-FDG) PET/CT scan.

**Fig. 1:**
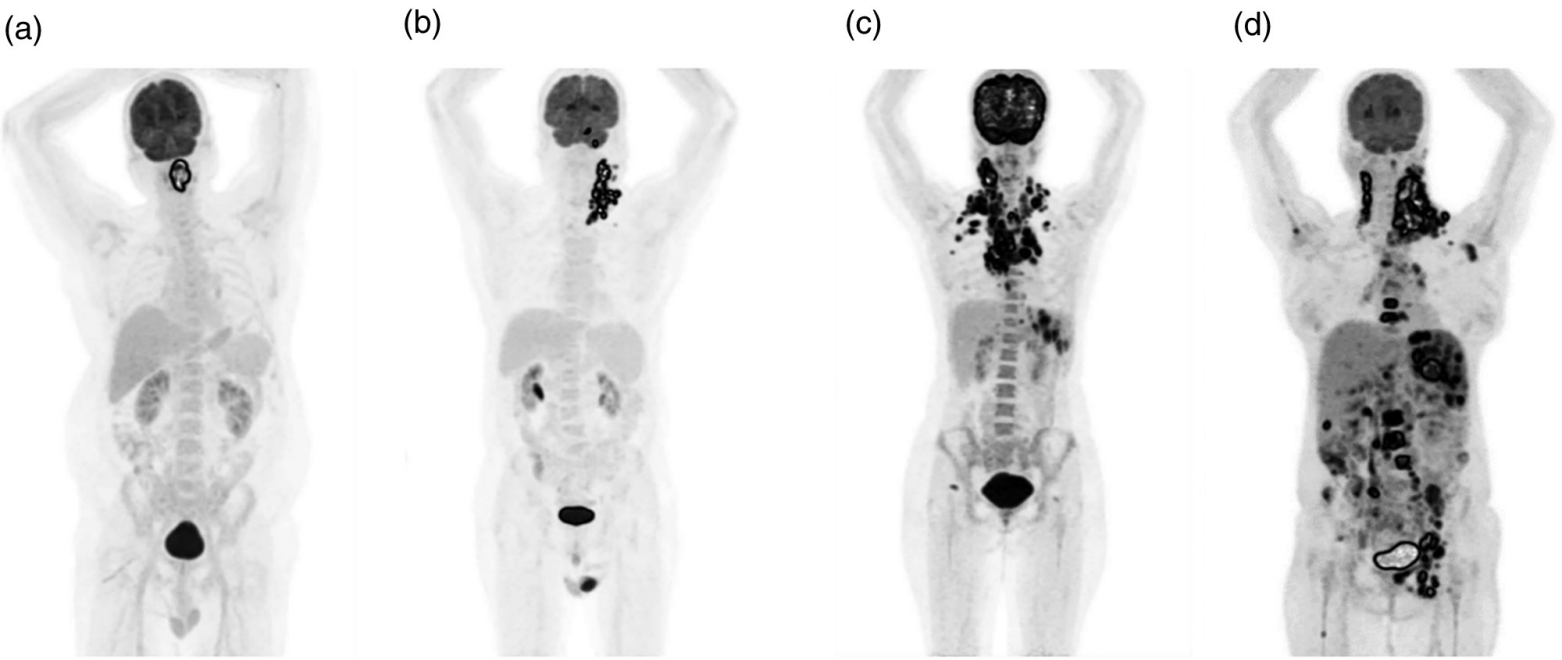
Maximum intensity projection images of patients with different disease stages: (a) DLBCL stage 1, (b) DLBCL stage 2, (c) nodular sclerosis stage 3, and (d) nodular sclerosis stage 4. DLBCL, diffuse large B-cell lymphoma.

The two centers’ patients varied greatly in terms of geography and ethnicity. At center 2 (Razavi Hospital), more than 80% of the patients were Arab, and the remaining patients were from northeastern Iran, where the majority of the patients were also Arab. At center 1 (Masih Daneshvari Hospital), on the other hand, over 95% of the patients were of Iranian ancestry, mostly from the country’s central areas (Fig. 1).

### Imaging protocol

#### Center 1

The PET/CT examinations were conducted on a GE Discovery 690 scanner, which has a 64-slice CT and time-of-flight technology. Whole-body scans were conducted from the top of the head (vertex) to the mid-thigh. The VUE Point HD/FX technique was used for image reconstruction. The average uptake time was 60 min, with individual uptake times ranging from 45 to 75 min. The PET scans took 2–3 min for each PET imaging bed position, and the PET scans maintained a slice thickness of 3.75 mm, while the low-dose CT scans had slice thicknesses of 1.33–2.5 mm. The Smart milliampere-seconds algorithm was used to automatically adjust the X-ray tube current based on the patient’s weight, with settings ranging from 50 to 150 mA. The helical pitch factor was continuously kept at 0.9, and the tube voltage was fixed at 120 kVp. Using information from the CT scans, the combined ^18^F-FDG PET/CT images were adjusted for attenuation and scattering.

#### Center 2

Whole-body PET/CT scans were conducted using the Biograph 6 TrueV scanner an hour after an intravenous dose of ^18^F-FDG was given, with uptake times varying from 50 to 70 min. According to the injection procedure, 0.1 mCi of ^18^F-FDG was administered for each kilogram of body weight. For 2.2 min, each PET scanning bed position was scanned. The pitch factor for the CT scan was 0.55. While the low-dose CT scans had slice thicknesses varying from 3 to 5 mm, the PET scans maintained a slice thickness of 5 mm. Filtered back projection was used to reconstruct CT images. The iterative ordered subset expectation maximization approach was used with 21 subsets and two iterations for PET scans. In addition, attenuation correction and scatter correction were applied to each image.

### Patient selection criteria

Patients with incomplete imaging data, those without histopathological confirmation, those who had undergone treatment before the PET/CT scan, those with negative or inconclusive diagnostic results, those with suspected concurrent infections, those with liver cirrhosis or fibrosis that could impair normal liver uptake, those with recent or concurrent malignancies, including breast cancer, and those whose PET/CT images were compromised by artifacts or poor image quality were all excluded from the study.

### Primary image evaluation and lesion segmentation

To determine the exclusion criteria, a nuclear medicine physician with over 10 years of experience initially assessed the PET/CT images. In center 1, ‘The same physician assigned a stage based on the Lugano classification [2,35], which was then confirmed by a radiologist with up to 34 years of experience’. In center 2, another nuclear medicine physician independently evaluated the lymphoma stage without input from the experts at center 1. Lesions were subsequently delineated by a nuclear medicine doctor at center 1 using a semiautomated graph-based segmentation method [36], implemented as an add-on to the 3D Slicer software [37]. Manual adjustments to lesion boundaries were made as necessary.

Moreover, we segmented at least one lesion from each lymphatic and extra-lymphatic region; however, the number of tumors per patient was not limited, and in some cases, over 50 tumors were segmented from a single patient. Following lesion delineation, a three-dimensional region of interest with three diameters was defined in the liver’s right lobe, making sure it was lesion-free, in compliance with the PERCIST criteria [38], to compute tumor-to-liver ratio (TLR) radiomics.

### Image preprocessing and feature extraction

Resampling the images to achieve isotropic voxel spacing was the first step in the image analysis process. Standardized uptake value (SUV) maps were then generated using PET images. According to the guidelines established by the Image Biomarker Standardization Initiative, SUV maps were discretized using a fixed bin size of 0.25 SUV [27]. The 3D Slicer program’s PyRadiomics extension was used for this procedure [25]. In addition, features were extracted from the liver volume of interest on PET images, excluding the shape features, in order to compute the TLR of feature values. Because our method was patient-based and each patient had multiple tumors, we used statistical measures like mean, minimum, maximum, and median to aggregate the values of each feature across all of the patient’s lesions.

### Feature selection and machine learning elaboration

To minimize any center-related differences, we started by using ComBat to harmonize the data from various centers. To assess the impact of ComBat harmonization on reducing center-specific variability, we performed Kolmogorov–Smirnov tests to compare the distributions of radiomic features between two centers before and after harmonization. The Kolmogorov–Smirnov tests were conducted for all numeric features, and only those with sufficient valid samples in both centers were included in the analysis (a minimum of 10 samples per group). Missing values were imputed using mean imputation before testing. Features that initially showed significant intercenter differences (*P* < 0.05) and demonstrated the greatest improvement in *P* values postharmonization were identified and selected.

To enhance the generalizability of our models and mitigate center-specific biases, we employed a center-based data split, using center 1 for training and center 2 for external testing. This approach simulates real-world clinical scenarios where a model trained at one institution is applied to data from a different institution, reducing the risk of data leakage and ensuring robust performance across diverse imaging protocols and patient cohorts. The most important radiomics and clinical features were then found by performing feature selection using SelectKBest with an analysis of variance *F* test. Feature selection was performed using the SelectKBest method with the f_classif scoring function, integrated within each model's pipeline. The number of selected features (*k*) was tuned as a hyperparameter (*k* = 15, 20) during grid search, allowing each model [XGBoost (XGB), Logistic Regression, and Random Forest] to identify the optimal feature subset independently. This model-specific feature selection enhances comparability and robustness in feature importance analyses by tailoring the feature set to each classifier's characteristics. In addition, this univariate filtering approach indirectly reduces multicollinearity by prioritizing statistically informative and nonredundant features. Although explicit multicollinearity assessment (e.g. variance inflation factor) was not performed, the combined use of feature selection, feature scaling (StandardScaler), and class balancing (Synthetic Minority Over-sampling Technique) contributes to more stable model training, particularly for algorithms such as logistic regression that are sensitive to feature correlations. We used GridSearchCV to maximize the number of chosen features, ensuring optimal performance. Next, we used a pipeline that included feature selection and feature scaling with StandardScaler to train three machine learning models: logistic regression, random forest, and XGBoost. Using GridSearchCV, we adjusted hyperparameters for each model, including the number of estimators for random forest, the regularization strength for logistic regression, and the learning rate and maximum depth for XGB. We used stratified five-fold cross-validation on the training data to guarantee model robustness. We evaluated each predictor’s impact using the logistic regression coefficients. To determine which predictors had the greatest impact, we computed feature importance for Random Forest and XGB. Lastly, we used the independent test set from center 2 to validate the models, assessing their performance using sensitivity, specificity, accuracy, and area under the curve (AUC) receiver operating characteristic curves. With balanced accuracy serving as the main metric, we evaluated the model’s performance on the unseen data to determine which one performed best, paying particular attention to the sensitivity and specificity balance.

### Survival analysis

To demonstrate the clinical relevance of disease stage in patient prognosis, a Kaplan–Meier survival analysis was conducted to compare outcomes between patients with early-stage (stages I–II) and advanced-stage (stage III–IV) lymphoma. This analysis was performed only using data from center 1, as survival data from center 2 were not available. The survival distributions between the two groups were compared using the log-rank test, which gives equal weight to all time points, and the Gehan–Breslow–Wilcoxon test, which places greater emphasis on early events.

To estimate the relative risk of mortality associated with disease stage, hazard ratios and their corresponding 95% confidence intervals were calculated using both the Mantel–Haenszel and log-rank methods. Median survival times were estimated for each group. In cases where the median survival could not be calculated – such as in the early-stage group where many patients remained alive at last follow-up – it was reported as undefined. A *P* value of less than 0.05 was considered statistically significant.

## Results

### Patient demographics

As shown in Table 1, the cohort exhibited distinct demographic profiles when stratified by disease stage (early vs. advanced). Patients with advanced disease were markedly older than those in the early stage, with a mean age of 47.6 ± 20.3 vs. 39.4 ± 19.5 years (Δ = 8.2 years). A Shapiro–Wilk test confirmed nonnormal age distributions in both groups (advanced: *P* = 0.044, early: *P* = 0.005), prompting the use of the Mann–Whitney *U* test, which revealed a statistically significant age disparity (*U* test; *P* = 0.002). The size of this difference, with a Cohen’s *d* of 0.40, indicates that it is clinically significant, suggesting that age could play an important role in how the disease progresses.

**Table 1:** Demographic characteristics of patients stratified by disease stage (early vs. advanced) and data center (train vs. test)

Stage	Center	Patient number	Mean age ± SD	Male (*n*)	Female (*n*)	Mean weight ± SD
Advance	2-Test set	51	51.73 ± 18.73	31	20	69.86 ± 14.92
Advance	1-Train set	69	44.48 ± 21.01	40	29	69.58 ± 18.28
Early	2-Test set	32	44.56 ± 20.81	18	14	70.28 ± 17.10
Early	1-Train set	89	37.58 ± 18.74	54	35	70.46 ± 19.20

A pronounced male predominance persisted across stages, though more prominent in the advanced stage (59.2% male and 71 males vs. 49 females) compared to the early stage (56.2% male and 68 males vs. 53 females).

**Fig. 2:**
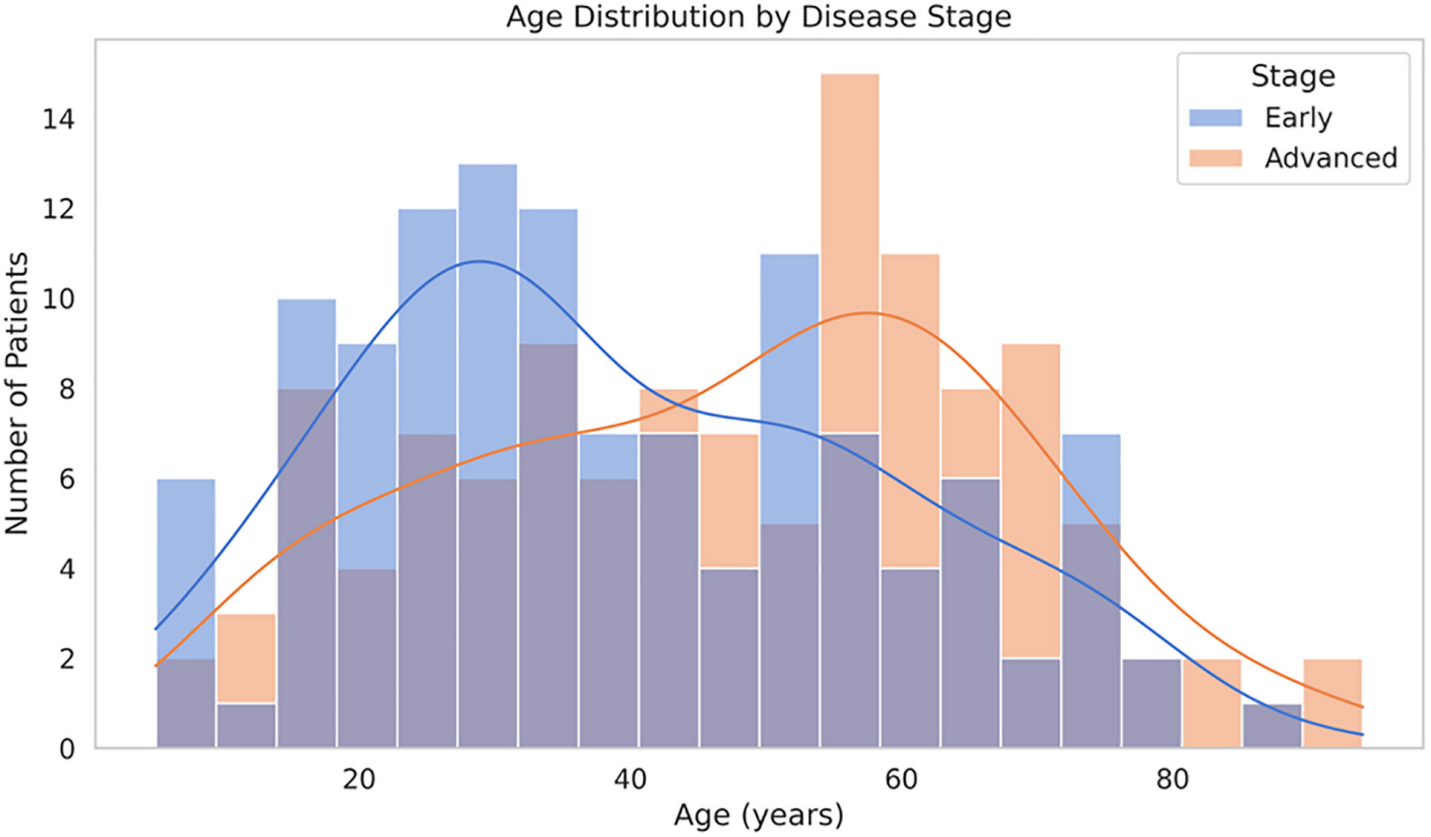
Histogram with kernel density estimates showing age distribution by disease stage.

As shown in Fig. 2, patients in the advanced stage are mostly older – typically between 40 and 60 years – while those in the early stage tend to be younger, often under 40. This matches earlier findings of an average age difference of about 8 years between the groups (advanced: 47.6 ± 20.3 vs. early: 39.4 ± 19.5), suggesting that age could be linked to disease severity.

However, because both groups span a wide age range (20–80 years), it is clear that age is not the only factor at play. The relatively large variation in ages (around 20 years) indicates that other influences – like genetics or underlying health conditions – might also be important. The slightly right-skewed distribution in the advanced group points to more older patients pushing up the severity curve, though the presence of younger patients in that group reminds us not to generalize. So, while age may be a meaningful risk factor, it should not be viewed in isolation. These findings support the need for routine screening across all age groups and a more personalized approach that includes other risk indicators.

**Fig. 3:**
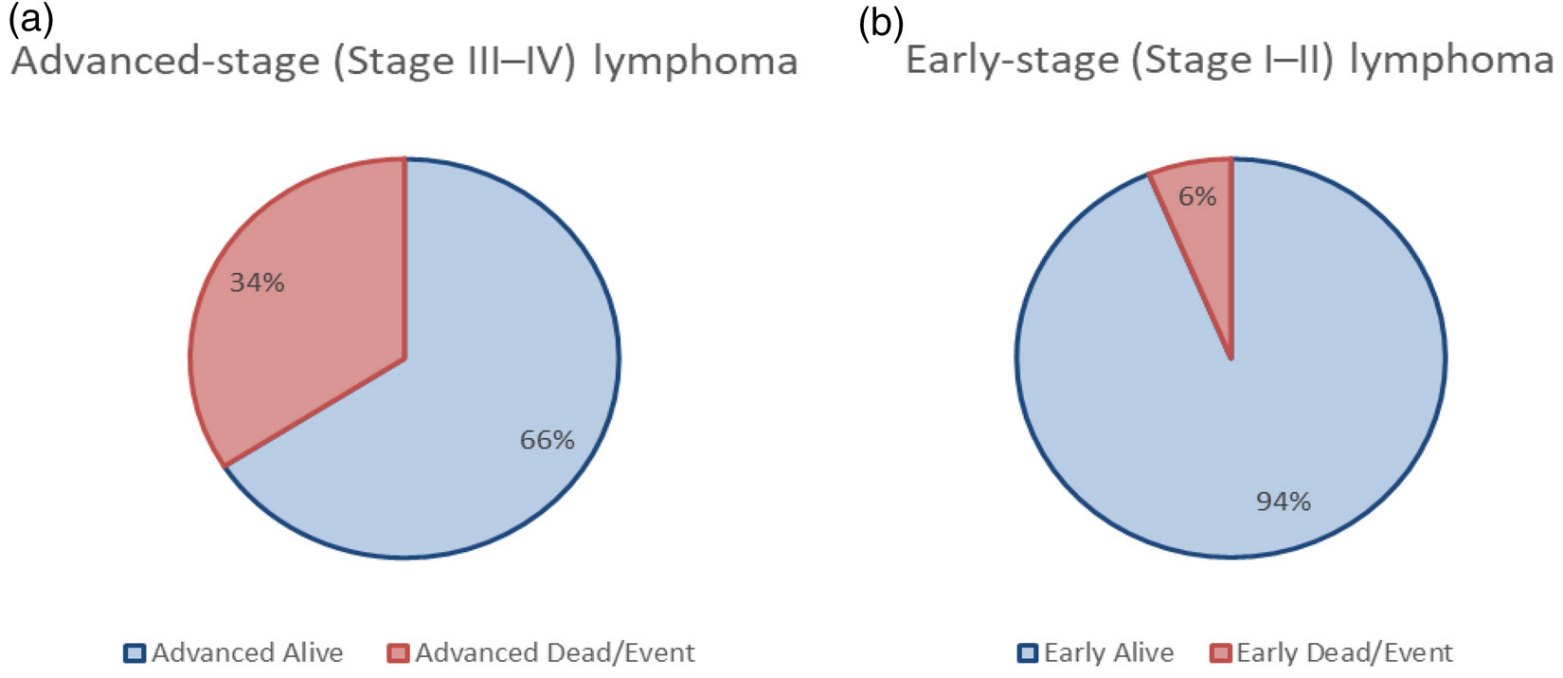
Proportion of survivors and deceased patients in each group: (a) advanced stage and (b) early stage.

Figure 3 compares the survival outcomes for patients with lymphoma in early and advanced stages (I–II vs. III–IV). Panel a shows the proportion of survivors and deceased patients in the advanced-stage group, while panel b does the same for early-stage patients. The graph shows that advanced-stage patients have a lower survival rate, which is common since more advanced stages generally lead to worse outcomes. The differences in survival rates could be due to how the disease responds to treatment or its level of aggressiveness at different stages.

### Harmonization

**Fig. 4:**
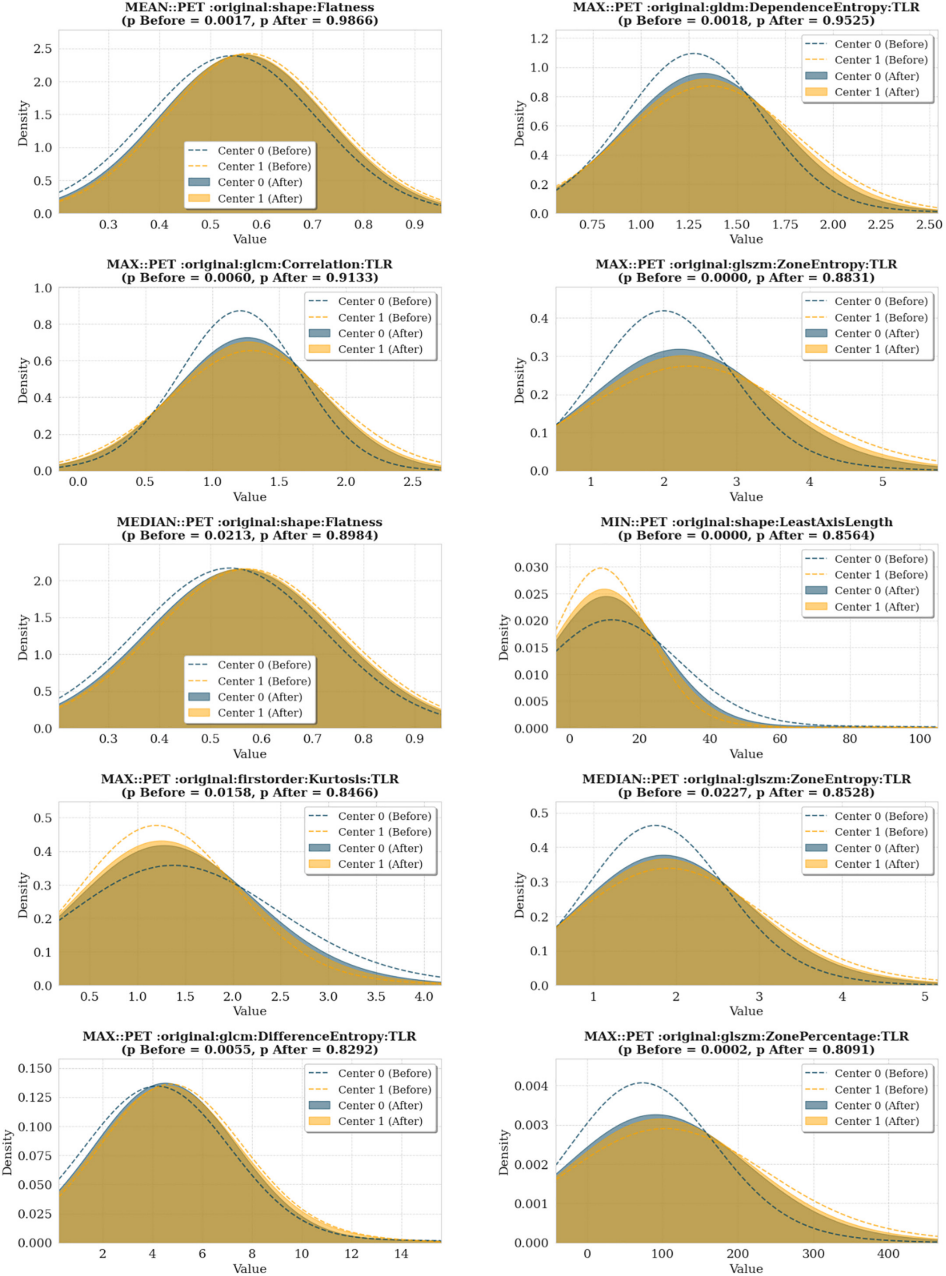
Kernel density estimation plots of selected radiomic features from the combined nodal and extranodal data before and after ComBat harmonization. Dashed lines represent feature distributions before harmonization (dark blue: center 0, orange: center 1), while shaded areas indicate distributions after harmonization (dark blue: center 0, orange: center 1).

**Fig. 5:**
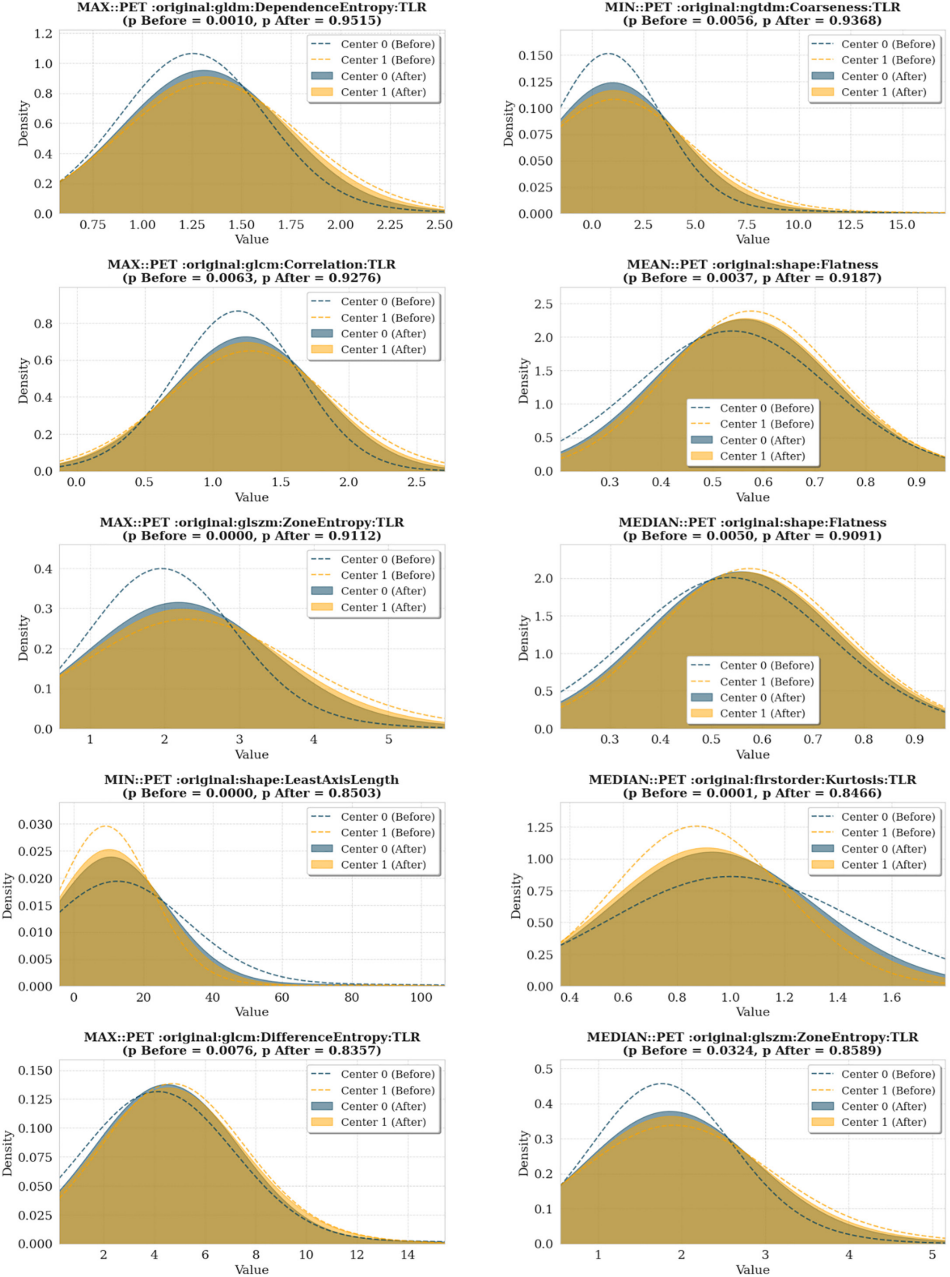
Kernel density estimation plots of selected radiomic features from nodal data before and after ComBat harmonization. Dashed lines represent feature distributions before harmonization (dark blue: center 0, orange: center 1), while shaded areas indicate distributions after harmonization (dark blue: center 0, orange: center 1). TLR, tumor-to-liver ratio.

To visually assess the distributional differences between centers before and after harmonization, kernel density estimation plots were generated for the top features with the most significant Kolmogorov–Smirnov statistics (Figs. 4 and 5). These plots demonstrate that ComBat effectively reduced intercenter differences in most features. A full list of Kolmogorov–Smirnov statistics for all features is provided in Supplementary Table S1, Supplemental digital content 1, https://links.lww.com/NMC/A353.

### Radiomics-demographic combined model

The results of different machine learning models for classifying early- vs. advanced-stage lymphoma are shown in Table 2. As illustrated, adding extranodal radiomics to nodal ones improved the models’ performance. Logistic regression achieved an AUC of 0.87 after incorporating extranodal radiomics (a 0.13 increase), and sensitivity improved from 0.72 to 0.78. Random Forest’s AUC increased from 0.74 to 0.78, with sensitivity rising from 0.72 to 0.84. XGB’s AUC improved marginally from 0.74 to 0.75, while sensitivity increased from 0.75 to 0.78.

**Table 2:** Performance metrics of machine learning models (XGBoost, Logistic Regression, and Random Forest) for classifying early- vs. advanced-stage lymphoma based on radiomics features

		Nodal radiomics			(Nodal + extra nodal) radiomics	
Model	Metric	CV training (Mean ± SD)	External test	CV training (Mean ± SD)		External test
Logistic Regression	AUC	0.68 ± 0.09	0.74	0.73 ± 0.09		**0.87**
	Accuracy	0.63 ± 0.09	0.66	0.64 ± 0.08		**0.72**
	Sensitivity	0.64 ± 0.18	0.78	0.59 ± 0.15		**0.88**
	Specificity	0.61 ± 0.07	0.59	0.71 ± 0.04		**0.63**
XGB	AUC	0.60 ± 0.11	0.74	0.64 ± 0.14		0.75
	Accuracy	0.57 ± 0.12	0.66	0.59 ± 0.11		0.67
	Sensitivity	0.58 ± 0.18	0.75	0.56 ± 0.17		0.78
	Specificity	0.55 ± 0.06	0.61	0.62 ± 0.07		0.61
Random Forest	AUC	0.67 ± 0.11	**0.75**	0.72 ± 0.08		0.81
	Accuracy	0.63 ± 0.12	**0.66**	0.63 ± 0.08		0.67
	Sensitivity	0.61 ± 0.13	**0.72**	0.62 ± 0.15		0.84
	Specificity	0.65 ± 0.14	**0.63**	0.65 ± 0.08		0.57

Metrics are reported for both CV and external test sets.

The bold values indicate the model with the best performance based on AUC in both internal and external tests.

AUC, area under the curve; CV, cross-validation; XGB, XGBoost.

**Fig. 6:**
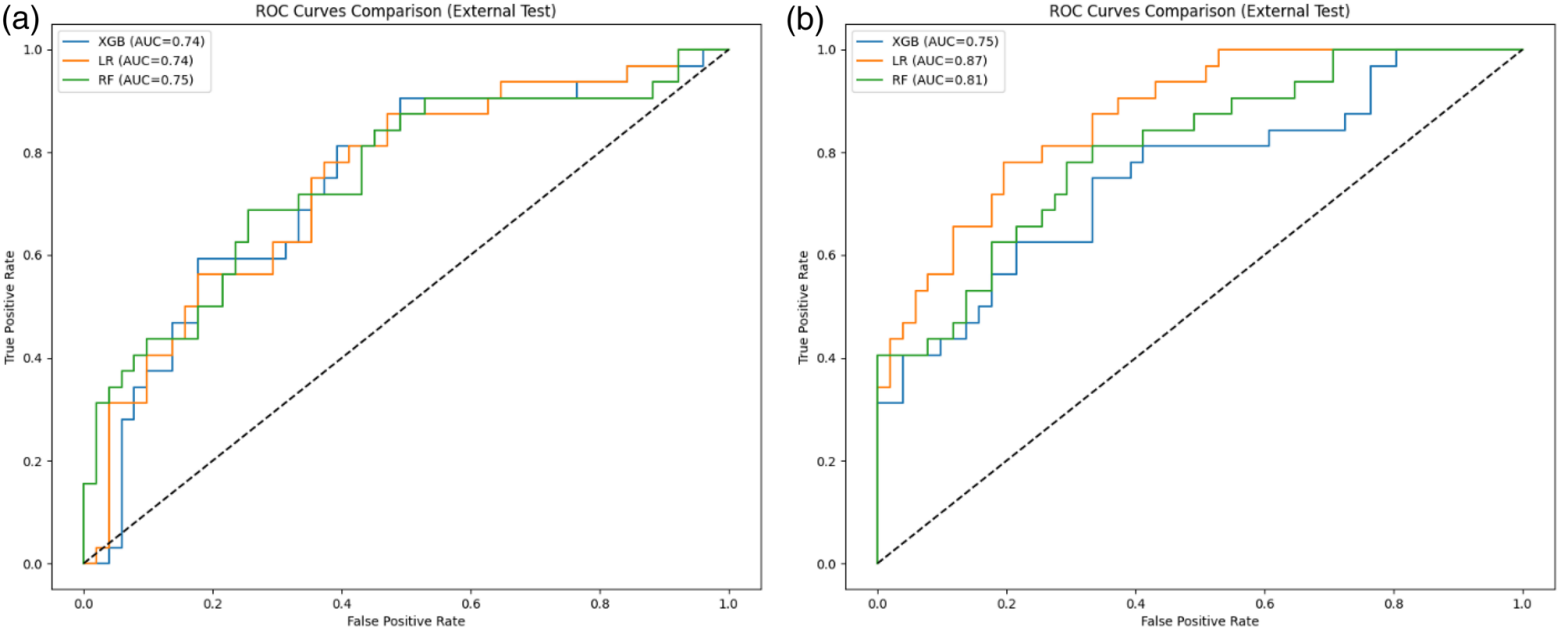
ROC curves of machine learning models (XGBoost, Logistic Regression, and Random Forest) for classifying early vs. advanced stage lymphoma. (a) Models using nodal radiomics only. (b) Models using combined nodal and extranodal. AUC, area under the curve; LR, Logistic Regression; RF, Random Forest; ROC, receiver operating characteristic; XGB, XGBoost.

Figure 6 illustrates the AUC values for each model, highlighting logistic regression with combined nodal and extranodal radiomics as the best-performing model with an AUC of 0.87 and a sensitivity of 0.88 on the external test set.

**Fig. 7:**
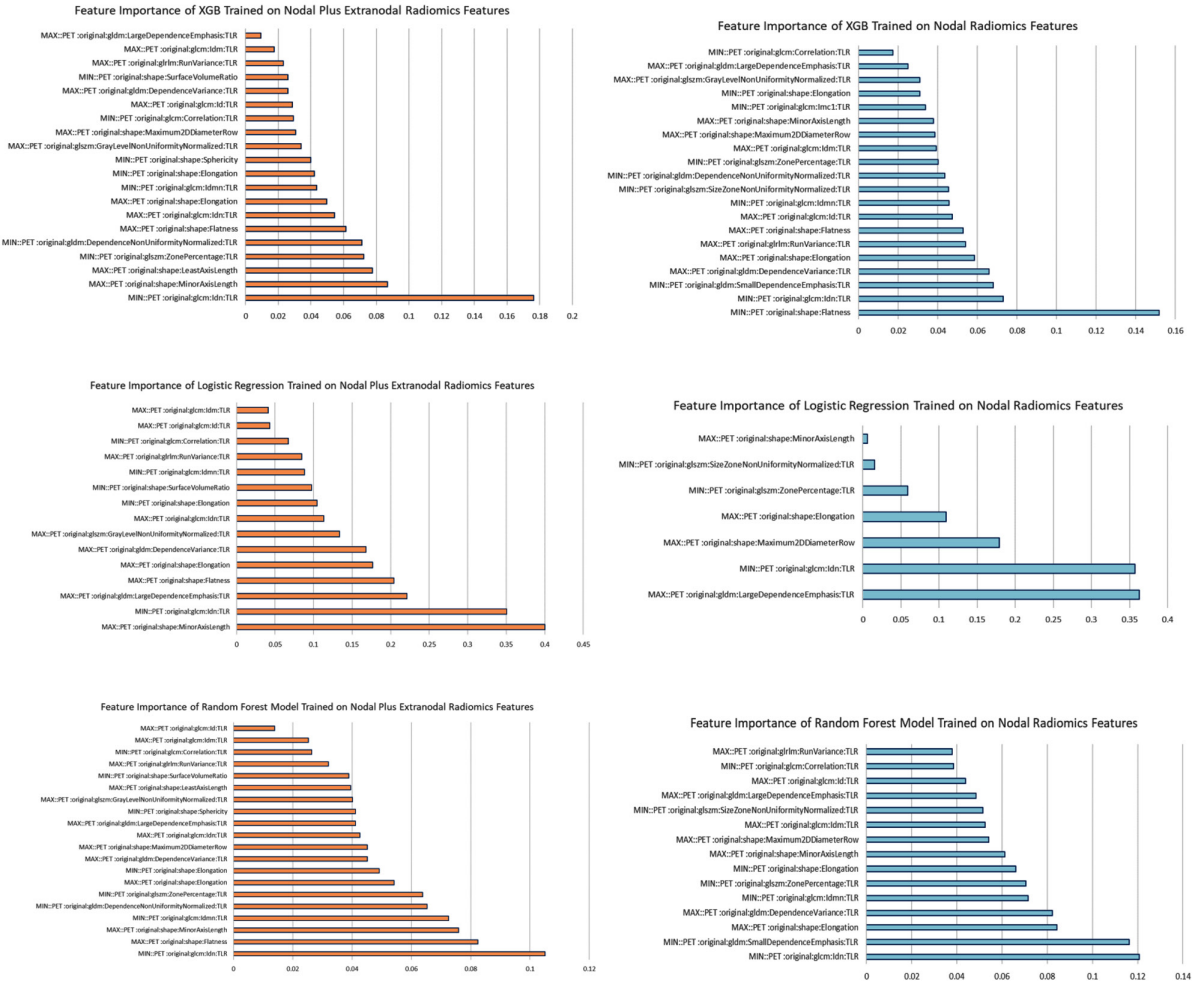
Feature importance plots for three machine learning models (XGBoost, Logistic Regression, and Random Forest) trained on nodal-only (blue) and combined nodal plus extranodal (orange) PET radiomics features. The importance of each feature is ranked by its contribution to the classification of early vs. advanced stage lymphoma. XGB, XGBoost.

As illustrated in Fig. 7, both shape- and texture-related features played key roles in distinguishing early- from advanced-stage lymphoma. When extranodal radiomics were added, shape features such as elongation gained higher importance – especially in the logistic regression model; however, texture features reflecting heterogeneity and complexity remained influential across all models. This suggests that the way tumors look and feel, both inside and beyond the lymph nodes, tells an important story about disease stage – one that can support doctors in making better-informed decisions for their patients.

### Survival analysis

**Fig. 8:**
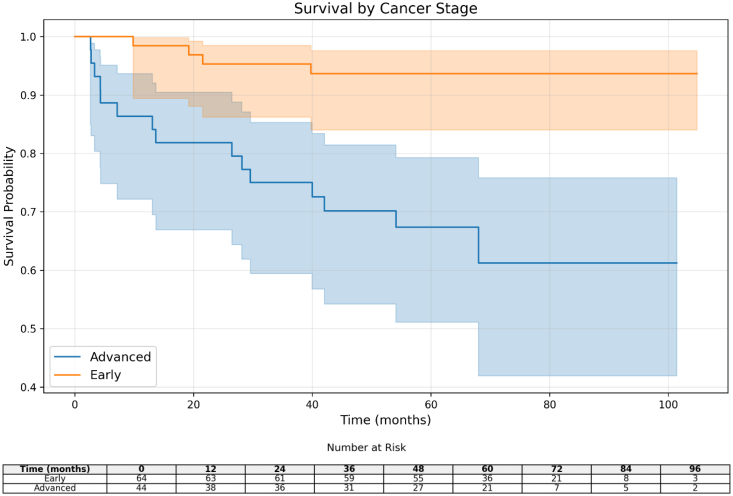
Kaplan–Meier survival curves comparing early-stage (orange) and advanced-stage (blue) cancer patients. Early-stage patients show significantly better survival (*P* = 0.0036).

As can be seen in the Kaplan–Meier plot (Fig. 8), patients with early-stage disease (orange) have significantly better survival than patients with advanced-stage disease (blue). The steeper slope of the curve in the advanced group indicates a higher mortality rate. This finding highlights the clinical importance of disease stage in patient prognosis.

Table 3 shows the results of the survival analysis between patients with early and advanced lymphoma. These results clearly demonstrate that the stage of the disease plays an important role in the prognosis of patients. The Lagrangian test, which was significant (*P* = 0.0036) and the low hazard ratios (about 0.22–0.26), indicate that the risk of death in advanced stage patients is about four times higher than in early stage patients. The lack of a specific number for the median survival in the early stage group indicates that these patients usually live longer, while the median survival of 84.27 units in the advanced stage indicates poorer outcomes in these patients. These results emphasize the importance of using more powerful treatments for advanced-stage patients, as well as the importance of early diagnosis and long-term follow-up to detect disease recurrence. Overall, the stage of the disease is one of the main factors in treatment decision-making and the prediction of the course of the disease.

**Table 3:** Summary of survival analysis results

Test/measure	Result
Log-rank test	*χ*² = 8.480, *df* = 1, *P* = 0.0036
Gehan–Breslow–Wilcoxon	*χ*² = 1.976, *df* = 1, *P* = 0.1598 (ns)
Median survival (days)	Early: undefined Advanced: 84.27
HR (Mantel–Haenszel)	Early/advanced: 0.2609 (95% CI: 0.1056–0.6445) Advanced/early: 3.833 (95% CI: 1.552–9.470)
HR (log-rank)	Early/advanced: 0.2247 (95% CI: 0.09113–0.5542) Advanced/early: 4.450 (95% CI: 1.804–10.97)

Comparison of survival between early and advanced groups using log-rank and Gehan–Breslow–Wilcoxon tests. HRs with 95% CI are reported.

*χ*², Chi-square; CI, confidence interval; *df*, degrees of freedom; HR, hazard ratio; ns, not significant; *P, P* value.

## Discussion

This study aimed to investigate the potential of radiomics-based machine learning in differentiating early stage vs. advanced stage in lymphoma, to replace the Lugano staging system currently used by doctors in clinical settings. We used two approaches: nodal radiomics alone, and a combination of nodal and extranodal radiomics, to see whether adding the second could bring any advantages.

To the best of our knowledge, this is the first study to use a radiomics-based machine learning approach for staging lymphoma. It integrates both nodal and extranodal imaging features. In other cancers, radiomics has already shown promising results for staging. For example, in lung cancer, a CT-based model achieved 77.5% precision and 78.7% recall in distinguishing early (stages I–II) from advanced (stages III–IV) cases [39]. In esophageal squamous cell carcinoma, radiomics models reached AUCs of 0.795 and 0.762 in the primary and validation cohorts, respectively [40]; however, no radiomics-based staging method has yet been developed for lymphoma.

The study was conducted across multiple centers, including diverse racial backgrounds, which enhances the generalizability of the findings. We achieved an AUC of 0.87 and a sensitivity of 0.88 with the best model that included both nodal and extranodal features on the external test set, highlighting the benefit of adding extranodal data to the model. On the other hand, the best model based only on nodal radiomics reached an AUC of 0.75 and a sensitivity of 0.72 on the same external test set; however, both models showed low specificity (0.63), which might be because of a few reasons. One possible explanation is that some imaging features in early and advanced stages can look quite similar, confusing the model. Another reason might be data imbalance or limited representation of certain extranodal patterns in the dataset. The use of an external test set emphasizes the robustness and external validity of the results.

In our analysis, some extranodal features, especially shape and texture ones, were among the most important in the model. This shows that these areas can provide extra information beyond just lymph node involvement. Because extranodal spread is often used in the Lugano system to define advanced stages, it makes sense that including these features improves the model’s performance. Also, some of the top features selected by the model – like shape irregularity and heterogeneity – have been linked to worse outcomes and survival in other studies. So, these radiomic features might not only help with staging, but could also be useful for predicting prognosis in the future.

Moreover, our survival analysis confirmed the strong prognostic value of disease stage. Patients with advanced-stage lymphoma had a nearly fourfold higher risk of death (hazard ratio: 0.22–0.26, *P* = 0.0036), and their median survival was around 84 months. These results highlight the importance of accurate staging in guiding treatment intensity and improving patient outcomes.

Our findings suggest that combining nodal and extranodal radiomics could be a game-changer for lymphoma staging. This approach offers a more objective and precise way to differentiate between early and advanced stages, which could potentially replace the traditional visual assessment methods used by doctors. The use of machine learning with radiomics not only removes subjectivity but also makes the process faster and more consistent, reducing human error. By relying on this technology, doctors could have a more reliable tool for staging, which could lead to better treatment decisions and improved patient outcomes.

However, further validation on larger datasets is needed before this method can be widely adopted in clinical settings. To support clinical applicability, we extracted feature importance for each model (XGB, Logistic Regression, and Random Forest) using model-specific metrics (e.g. absolute coefficients for logistic regression, feature importances for tree-based models), as detailed in Supplementary Table S2, Supplemental digital content 2, https://links.lww.com/NMC/A354. These results highlight the radiomic features most influential in distinguishing Hodgkin's lymphoma from non-Hodgkin lymphoma, which consistently showed high importance across models; however, to further enhance model transparency for clinical adoption, future work could incorporate explainable Artificial intelligence techniques such as SHapley Additive exPlanations or Local Interpretable Model-agnostic Explanations. These methods provide detailed insights into individual feature contributions and their impact on specific predictions, potentially increasing trust and interpretability among clinicians. Integrating such tools into clinical decision support systems could facilitate the translation of our radiomics-based approach into routine lymphoma staging workflows.

Intercenter variability remains a major challenge for the clinical adoption of radiomics-based models, particularly in multi-institutional studies where imaging protocols often differ. In our study, we addressed this issue using ComBat harmonization to reduce scanner- and protocol-induced biases in radiomic features. The results support ComBat’s utility in enhancing the reproducibility and generalizability of radiomics models across centers. While the majority of features showed substantial distributional alignment postharmonization, a few remained significantly different, likely because of underlying biological heterogeneity or persistent technical inconsistencies. These residual differences highlight the potential limitations of statistical harmonization alone. Future studies could benefit from combining ComBat with acquisition-aware harmonization techniques or leveraging imaging metadata directly within the modeling process to further improve robustness in multicenter settings.

### Conclusion

Our findings suggest that radiomics-based machine learning can offer a practical and reliable way to automate lymphoma staging based on the Lugano system – without relying on subjective visual assessments. By combining nodal and extranodal features, the model not only improves accuracy but also speeds up the process, which is especially helpful in busy clinical environments. With further validation and better interpretability, this approach could become a valuable tool to support faster and more consistent staging in everyday practice.

## Acknowledgements

Conception and design, acquisition of data, methodology, programming and data processing, analysis/interpretation of data, statistical analysis, literature search, and writing of the manuscript: S.H. Supervision, conception and design: S.M.R.A. Conception and design, supervision, edit manuscript: A.A.A. Data cleaning: M.C. Acquisition of data: H.V.Acquisition of data: F.E. and M.B.K. Conception and design, supervision, editing manuscript, final revision: H.A.

The research was approved by the Medical Ethical Review Committee of Shahid Beheshti University of Medical Sciences under the ethical code IR.SBMU.NRITLD.REC.1402.060. Informed consent from all participants was waived by the Medical Ethics Review Committee due to the noninterventional/retrospective design of the study.

The corresponding author may provide the data and materials used in this study upon reasonable request.

### Conflicts of interest

There are no conflicts of interest.

## Supplementary Material




